# Poly(*N*-isopropylacrylamide-*co*-2-((diethylamino)methyl)-4-formyl-6-methoxyphenyl acrylate) Environmental Functional Copolymers: Synthesis, Characterizations, and Grafting with Amino Acids

**DOI:** 10.3390/biom8040138

**Published:** 2018-11-06

**Authors:** Momen S. A. Abdelaty

**Affiliations:** Polymer and Biopolymer Lap, Department of Biology, Collage of Haql, Faculty of Science, University of Tabuk, Tabuk 71491, Saudi Arabia; momen@ut.edu.sa or abdelatymomen@yahoo.com; Tel.: +966-530049575

**Keywords:** environmental polymers, functional polymers, amino acids, grafted polymerization, Schiff’s base

## Abstract

Vanillin was used to synthesize a new derivative with an active aldehyde group and response to pH. It is named 2-((diethylamino) methyl)-4-formyl-6-methoxyphenyl acrylate, abbreviated to DEAMVA. The chemical structures were evaluated by ^1^H, ^13^C nuclear magnetic resonance (NMR), infrared (IR), and UV-Vis-spectroscopy, and all results demonstrated good statement. In order to achieve the dual responsive behavior thermo-pH with functionality, free radical polymerization of *N*-isopropylacrylamide with DEAMVA in different molar ratios (5, 10, 15 mol%) has been used, with azobisisobutyronitrile (AIBN) as the initiator. The chemical structure of the polymers was investigated by ^1^H NMR and IR. The dual responsive functional copolymer was exposed to a grafted process with tryptophan and tyrosine, both of which were also evaluated by ^1^HNMR and IR. Copolymers before and after grafting were physically investigated by size exclusion chromatography (SEC) for estimation of the molecular weight, the glass transition temperature by differential scanning calorimeter (DSC) and scanning electron microscope (SEM) for the surface morphology. The phase separation or lower critical solution temperature (LCST) (*T_c_*) of the polymer solution was determined not only by a turbidity method using the change in the transmittance with temperature, but also by micro-DSC. The conversion to an amino acid-grafted polymer was detected through Beer’s law for the absorption of the –CH=N- imine group by UV-Vis-Spectroscopy.

## 1. Introduction

In nature, many living creatures can change their behavior according to the surrounding environment; examples include the Venus flytrap (closes fast enough to catch its prey), the leaves of *Mimosa pudica* (collapses immediately when touched), sunflowers (follow the movement of the sun), sea cucumbers (change their stiffness in the face of danger), and chameleons (change color according to the nature of the environment) [1,2,3,4].

Over the past few years, many scientists have focused their interest on a new kind of polymer material with special characterizations that can develop its behavior with the surrounding environment. Several definitions were used like environment, intelligent, smart, and responsive, according to the physical/chemical properties. These polymer materials can develop their responses to temperature, the intensity of light, humidity, pH, ionic strength, and electric/magnetic fields. Basically, they can be classified as one of single stimuli-responsive, dual stimuli-responsive, or multistimuli responsive [5,6,7].

Temperature or Thermo-responsive polymer exhibits a critical solution temperature at which the polymer solution exposed to a phase separation with temperature; this can be attributed to intra- and inter-molecular interaction, or to a collapse in the polymer chain. Thermo-responsive water-soluble polymers exhibit two kinds of phase separation: (a) lower critical solution temperature (LCST), at which the separation occurs from a soluble (monophasic) to an insoluble state (biphasic) with raising temperature, and (b) upper critical solution temperature (UCST), which is the opposite of LCST. The most familiar thermo-responsive, water-soluble polymer is poly (*N*-Isopropylacrylamide) (PNIPAAm) demonstrated LCST of 32 °C. Notably, its mechanism and chemical composition are responsible for increasing its hydrophobicity (collapsed of polymer chain) or hydrophilicity (expansion of the polymer chain) [8,9,10].

On the other hand, polymers with acidic or basic groups in the main chain which facilitate the donation or withdrawal of protons are said to be “pH stimuli-responsive” or “pH environmental polymers”. The change of pH in a polymer solution leads to ionization and electrostatic interaction inducing collapse or expansion of the polymer chain. Poly (acrylic acid) (PAA) is a typical example of a polyacid; with pKa ≈ 5, it donates its protons and swells under basic conditions, while poly(*N*,*N*-dimethyl aminoethyl methacrylate) (PDEAEMA) is the most popular polybase; it accepts protons under acidic conditions, and expands due to Coulomb repulsion [11,12].

Dual responsive polymers can be prepared by copolymerization between two responsive monomers with different stimuli. The first dual responsive polymer was published by Kungwatchakun et al. in 1988 for thermal and light stimuli. Thermal and pH dual stimuli response have attracted a great attention in the field of drug delivery, sensors, actuators, and bio-separation [13,14,15,16,17,18,19]. The presence of a functional group is capable of forming ionic groups by dissociation or association upon protonation, which are incorporated into the backbone chain of the LCST polymer such as NIPAAm with a pH-responsive with ionizable groups, such as poly (acrylic acid) (PAA) or poly (*N*,*N*-dimethylaminoethylmethacrylate) (PDEAEMA) [20,21].

Vanillin has been used for the preparation of many kinds of monomers as a renewable resource and nontoxic material. Many researchers have used vanillin in the synthesis of bio-based monomers and polymers [22,23]. Several monomers and their polymers have been prepared using vanillin and its chemical modification of aldehyde, hydroxyl or both functional groups [24,25]. A new study has been reported by Audie K. Thompson et al. demonstrating the synthesis of hydrovanilloin by electrochemical dimerization of vanillin. This compound has been used as a renewable substitute for bisphenol A for the preparation of epoxy resin [26]. Moreover, aldehyde functional polymers and hydrogels have been widely used to couple with protein and peptides [27,28,29,30,31].

Due to the chemical structure of amino acids containing both of –COOH and –NH_2_ functional groups, their acrylate monomers could be prepared using the terminus carbon or nitrogen. The modification using terminus nitrogen was reported in a recent review [32]. On other hand, the C-terminus has been introduced by many authors to couple vinyl acrylate to free amine groups [32,33,34]. In 2014, Saswati Ghosh Roy and Priyadarsi De published an interesting review of pH-responsive polymers with amino acids in the side chains, and their potential applications [35]. The applications of side-chain amino acid-based monomers and polymers have been widely used in bio-separation, bio-membranes, drug delivery, and gene delivery [36,37,38].

This article orbits around the preparation of environmental polymers with response to temperature and pH which can be used for the bio-separation of amino acids by click reaction. Here, we used tertiary amine functional vanillin acrylate, which differs from our previously studied compounds because of its basic condition that facilitates the formation of Schiff’s base.

## 2. Material and Methods

### 2.1. Materials

Amino acids tyrosine and tryptophan (99% and 97% Acros, Düsseldorf, Germany), (AIBN, 98% Acrōs Germany) 2,2′-azobis(isobutyronitrile) were recrystallized from methanol, and *N*-isopropylacrylamide (NIPAAm, Düsseldorf) was recrystallized from distilled hexane. Vanillin (99% Düsseldorf, Germany), triethylamine (99% Merck, Darmstadt, Germany), acryloyl chloride (98% Merck, Darmstadt, Germany), formaldehyde (38% Sigma-Aldrich, Darmstadt, Germany), diethyl amine (99% Acros, Düsseldorf, Germany), Dichloromethane, dioxane, tetrahydrofuran (THF), and diethyl ether were distilled over potassium hydroxide. Other chemicals were used as received. For pH 1.68, pH 7 and pH 12.46 buffer solutions were used as purchased from Thermo Fisher (Loughborough, US).

### 2.2. Instrumentations and Measurements

^1^H (500 MHz) and ^13^C (125 MHz) NMR spectra were recorded on Bruker AV (Germany, Karlsruhe) in *d*^6^-DMSO or CDCl_3_. IR spectra were measured on a Vertex 70 FT-IR-4100 spectrophotometer (Germany, Karlsruhe). The samples were milled with dry potassium bromide KBr (99% Merck, Germany, Darmstadt) and pressed into pellets. The molecular weights number average molecular (Mn), weight average molecular weights (Mw), and polydispersity (Ð) were determined by gel permeation chromatography (GPC) on TSK gel α-3000, using a solution of LiBr (10 mM) in *N*,*N*-dimethylformamide (DMF) as an eluent at a flow rate of 1.0 mL/min, calibrated by polystyrene standards at 30 °C. This was used to determine the conversion of amino acid grafted polymers. UV/vis spectrometer (Perkin Elmer Lambda 45, UK) with metal covet stand and water bath (Julabo F12, UK) with thermostat for injection of the water cycle, and cooling system was used to measure the phase transition temperature (LCST) (*T_c_*). Over manual thermostat (Temperatur-Messgerät Md 3040, Beckmann+Egle, UK) was also used to adjust the actual temperature inside the solution at 2 °C/min over a temperature range of 5 to 80 °C. The polymer solution was 1 wt % in water or pH solution. Definitional scanning calorimeter (micro-DSC) was used to determine the transition temperature (*T_c_*) of the polymer solution; the thermograms of the polymer solutions were recorded at a cooling and heating rate of 5 °C/min. A concentration of 50 mg/mL was dissolved in deionized water, and the transition temperature was determined as the onset value in the thermogram. The glass transition temperature of solid polymers was recorded by Perkin Elmer Differential Scanning Calorimeter (DSC) Pyris 1 (USA, Waltham, MA, USA) with a heating and cooling rate of 5 °C/min. The morphological feature of the polymer before and after grafting was examined by Scanning Electron Microscopy (SEM) using a Zeiss NEON 40 instrument (USA, San Diego, CA, USA); 2 kV (30 µm aperture) and Bal-Tec SCD 500 sputter (USA, San Diego, CA, USA) coated with a film thickness monitor QSG 100. We applied approx. 4 nm of gold-palladium (Au:Pd = 80:20). The melting point was recorded by Stuart Digital Melting Point Apparatus (UK, Staffordshire, ST15 OSA), temperature range: Ambient to 300 °C, temperature resolution: 1 °C, and Ramp rates: 20 °C per min to plateau, 2 °C per min to melt.

### 2.3. Synthesis of 2-((diethylamino)methyl)-4-formyl-6-methoxyphenyl acrylate (II) (DEMAVA)

#### 2.3.1. Step 1: Preparation of 3-((diethylamino)methyl)-4-hydroxy-5-methoxy-benzaldehyde) (**I**)

Equal amounts of (20 g, 0.13 mol) vanillin (4-hydroxy-3-methoxy benzaldehyde), (20 g, 0.66 mol) formaldehyde and (20 g, 0.27 mol) diethylamine were dissolved in 100 mL ethanol. The reaction mixture was stirred and refluxed with a water trap for 3 h in a 250 mL, single-neck, round-bottomed flask. Water was removed from the trap and the solution was allowed to cool at room temperature. The product was concentrated under reduced pressure and allowed to cool for a few hours at room temperature; it was then extracted as a solid material. It was dried overnight in a desiccator with calcium chloride, and fitted with reduced pressure. The product Yield was 97%. It comprised a yellowish white solid with a melting point (mp) of 126 °C.

^1^H NMR (500 MHz, CDCl_3_): δ (ppm) *=* 1.13 (t, 6H, 12), 2.66 (q, 4H, 11), 3.85 (s, 2H, 10), 3.90 (s, 3H, 9), 7.12 (d, *^4^J* = 1.6, 1H, 2), 7.30 (d, *^4^J* = 1.6 Hz, 1H, 4), 9.74 (s, 1H, 8).

^13^C-NMR (125 MHz, CDCl_3_): δ (ppm) *=* 11.02 (2C, 12), 46.37 (2C, 11), 55.94 (1C, 10), 56.51 (1C, 9),109.69 (1C, 2), 121.30 (1C, 4), 125.18 (1C, 5), 127.77 (1C, 3), 148.72 (1C, 6), 155.26 (1C, 1), 191.61 (1C, 8).

IR (KBr): ν (cm^−1^) = 2988 (s) (CH_2_, CH_3_), 1708 (s) (C=O), 1654 (s) (C=C), 869–823 (m) (Ar–CH).

#### 2.3.2. Step 2: Preparation of 2-((diethylamino)methyl)-4-formyl-6-methoxyphenyl acrylate) (**II**) (DEAMVA)

In a 500 mL, three-neck flask fitted with an argon balloon, refluxed condenser, and dropping funnel, (20.85 g, 0.087 mol) of 3-((diethylamino)methyl)-4-hydroxy-5-methoxy-benzaldehyde **(**DEAMV) (**I**) was dissolved in dry CH_2_Cl_2_ (300 mL). Pellets of sodium hydroxide (15.0 g, 0.375 mol) were added and the mixture was stirred. The overall reaction mixture was cooled in an ice bath to 0–5 °C. (8.1 g, 0.0885mol). Then, acryloyl chloride was dissolved in 50 mL dry CH_2_Cl_2_ and added dropwise by dropping funnel. The solution color changed from colorless to a yellowish suspension. After 1h, the ice bath was taken off and stirring was continued at room temperature (25–27 °C) for 8 h. The precipitate of sodium chloride was filtered, and then the filtrate was taken to a rotatory evaporator to remove the solvent and concentrate the crude product. The purification process was done by dissolving the crude product in CH_2_Cl_2_ and washing it three times with distilled water, once with 0.1 M Na_2_CO_3_, and again with distilled water. The pure product was dried by stirring in MgSO_4_ overnight; the solvent was removed under reduced pressure to collect the product, and decanted in a dry flask injected by nitrogen. Yield was 75%, comprising an orange viscous liquid.

^1^H NMR (500 MHz, CDCl_3_): δ (ppm) = 0.94 (t, 6H, 10), 2.44 (q, 4H, 9), 3,52 (s, 2H, 8), 3.82 (s, 3H, 11), 6.05 (dd, *^2^J* = 1.2 Hz, *^3^J* = 10.5 Hz, 1H, 14), 6.36 (dd, *^3^J* = 10.5 Hz, *^3^J* = 17.3 Hz, 1H, 13), 6.64 (dd, *^2^J* = 1.2 Hz, *^3^J* = 17.3 Hz, 1H, 14), 7.39 (d, *^4^J* = 1.7 Hz, 1H, 2), 7.66 (d, *^4^J* = 1.7 Hz, 1H, 3- or 4), 9.95 (s, 1H, 7).

^13^C-NMR (125 MHz, CDCl_3_): δ (ppm) = 11.69 (2C, 10), 47.00 (2C, 9), 51.12 (1C, 8), 56.12 (1C, 11), 108.62 (1C, 2), 127.18 (1C, 4), 127.18 (1C, 13), 134.52 (1C, 5), 134.82 (1C, 3), 134.9 (1C, 14), 143.43 (1C, 6), 152.08 (1C, 1), 162.96 (1C, 1), 191.42 (1C, 7).

IR (KBr): ν (cm^−1^) 2915, 2834 (s) (CH_2_, CH_3_), 1639 (s) (C=O), 1610 (s) (C=C), 866–825 (m) (Ar-CH).

### 2.4. Preparation of Poly (NIPAAm-co-DEAMVA) with 5, 10 and 15 mol % of DEAMVA (***IIIa–c***)

In a 100 mL, two neck, round bottom flask with in and outlet argon and 25 mL dropping funnel, 5, 10, and 15 mol %, 0.544 g and 1.088 g, 1.632 respectively of (**II**) was added to 2 g (0.0176 mol) NIPAAm in 50 mL absolute ethanol. The mixture was purged by nitrogen and stirred. After complete dissolving, AIBN was dissolved in 15 mL absolute ethanol and added as 10^−3^ mol % of the total mol% of monomers. The separating funnel was removed and the mixture was stirred in an oil bath at 65 °C for 8 h under an inert atmosphere. The polymerization process was stopped and the solution cooled at room temperature and then in a refrigerator for termination. The polymer was precipitated in diethyl ether, at −40 °C (liquid nitrogen + acetone in 1.9 L Dewar flask), then dissolved in THF, and re-precipitated in diethyl ether to remove the unreacted monomers and impurities. Yield: 88%, 82%, and 76% for 10%, 15% and 20% mol of **II** respectively, Physical state: Yellowish solid.

^1^H NMR (500 MHz, CDCl_3_): δ(ppm)= 0.74–1.37 (m, 12H, 7,13), 1.47–2.82 (m, 6H, 2,4, 1,3),2.56–2.78 (m, 4H, 13), 3.42–3.50 (m, 2H, 14), 3.60–3.73 (m, 3H, 8), 3.80–4.10 (br., 1H, 6), 5.85–6.90 (br., 1H, 5), 7.07–7.60 (m, 3H, 9,10), 9.73–10.1 (br. (s), 1H, 11).

IR (KBr): ν (cm^−1^) 2990 (s) (6, 7–CH–Aliphatic), 1714–1743 (s) (5–C=O), 1640–1650 (s) (1–C=O), 1134 (s) (12–OCH_3_).

### 2.5. Synthesis of Grafted 15 mol % Poly (NIPAAm-co-DEAMVA) with Tryptophan and Tyrosine (***IV–V***)

In a two-neck flask fitted with a reflux condenser with a water trap and rubber stopper to pass a syringe of inlet and outlet argon, a mixture of 1.0 g of 10 mol % Poly(NIPAAm-*co*-DEAMVA) and 1.0 g of (0.005 mol tryptophan, 0.055 mol tyrosine) was dissolved in 30 mL ethanol-water 50/50 V/V %. The reaction mixture was allowed to reflux and stirred for 8 h at 110–120 °C. The product was transported to a one neck flask and the solvent was evaporated under reduced pressure. The product was purified by dissolving in THF and reprecipitated in diethyl ether at −30 °C to avoid any unreacted molecules. Physical state: with **VI** (Tryptophan) Brownish solid, **V** (tyrosine) yellowish solid.

### 2.6. Synthesis of Grafted 15 mol % Poly (NIPAAm-co-DEAMVA)-g-tyrosine as a Function of Time

In order to study the conversion of polymer to the grafted polymer with amino acid, we repeated the previous reaction with tyrosine using the same method several times as a function of the reaction time (15, 180, 360, 840 min). The products were separated and purified. UV-Vis spectroscopy was used to determine the reaction conversion for each running reaction, according to the change of the absorbance intensity.

(NIPAAm-*co*-DEAMVA)-*g*-Tryptophan (**IV**):

^1^H NMR (500 MHz, DMSO): δ(ppm) = 0.75–1.26 (m, 12H, 5,18), 1.28–1.64 (m, 4H, 15), 1.87–2.25 (m, 2H, 14), 2.50–2.54 (m, 4H, 4), 2.95–3.05 (m, 2H, 8), 3.30–3.40 (m, 2H, 10), 3.50–3.70 (m, 4H, 2), 4–4.20 (m, 17), 6.08–6.29 (m, 1H, 11), 6.80–7.70 (m, 6H, 6,13), 8.47–8.53 (br.; (s), 1H, 7), 9.15–9.30 (s, 1H, 12).

IR (KBr): ν (cm^−1^) 2993 (m) (CH–Aliphatic), 1663–1650 (s) (12–C=O), 1574–1563 (s) (CH=N), 1030–1107 (s) (11–OCH_3_), 750–742 (s) (1–4–CH–aromatic).

P(NIPAAm-*co*-DEAMVA)-*g*-tyrosine (**V**)

^1^H NMR (500 MHz, DMSO): δ(ppm) 0.44–0.75 (m, 6H, 7), 0.76–1.11 (m, 6H, 16), 1.12–1.89 (m, 6H, 1–4 repeating unit), 2.32–2.45 (m, 2H, 12), 2.34–2.44 (m, 1H, 6), 3.48–3.68 (m, 2H, 5), 3.75–3.78 (m, 3H, 9), 3.95–4.22 (m, 1H, 15),4.23–4.25 (m, 1H, 11), 4.89–5.13 (m, 1H, 5),6.30–7.95 (m, 8H, 8, 13), 8.12–8.21 (br. (s), 1H, 10).

IR (KBr): ν (cm^−1^) 2990 (m) (CH–Aliphatic), 1655–1660 (s) (C=O), 1570–1560 (s) (–CH=N), 1030–1107 (s) (–OCH_3_).

## 3. Results and Discussion

### 3.1. Synthesis of Monomer, Copolymers and Grafted Copolymers

**Figure 1:**
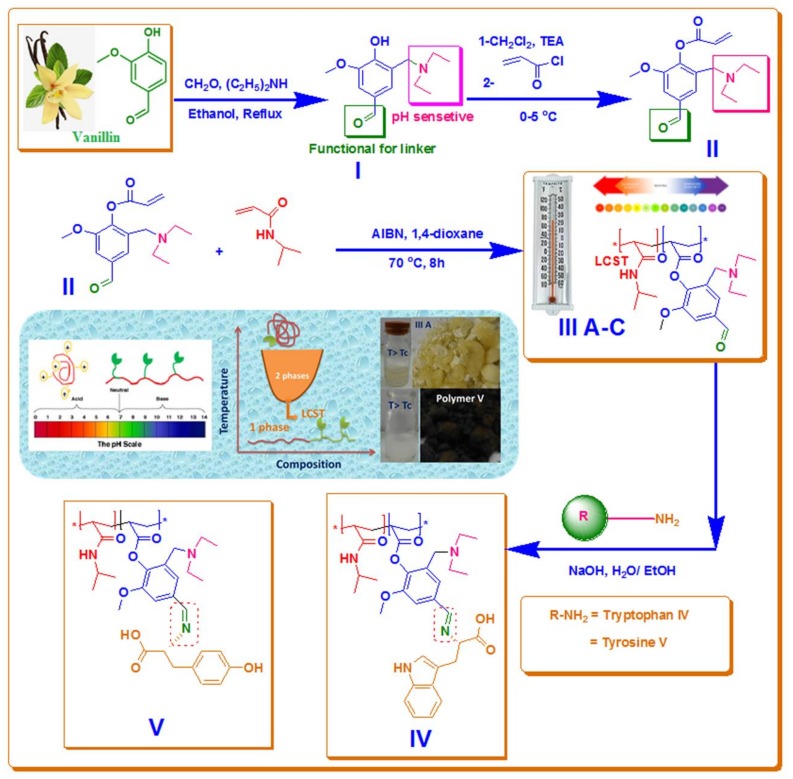
Synthesis of DEAMVA, copolymers and grafted polymers with NIPAAm.

Monomer, copolymers, and grafted copolymers were synthesized according to the chemical conditions described in Figure 1. In the present study, we used a vanillin compound for the synthesis of new kinds of acrylate monomers. The new monomer (**II**) [(diethylamino) methyl]-4-formyl-6-methoxyphenyl acrylate (DEMAVA) was synthesized in two steps, as described in Figure 1.

#### 3.1.1. Step 1: Is the formation of (3-[(diethylamino) methyl)-4-hydroxy-5-methoxy-benzaldehyde) (**I**)

**Figure 2:**
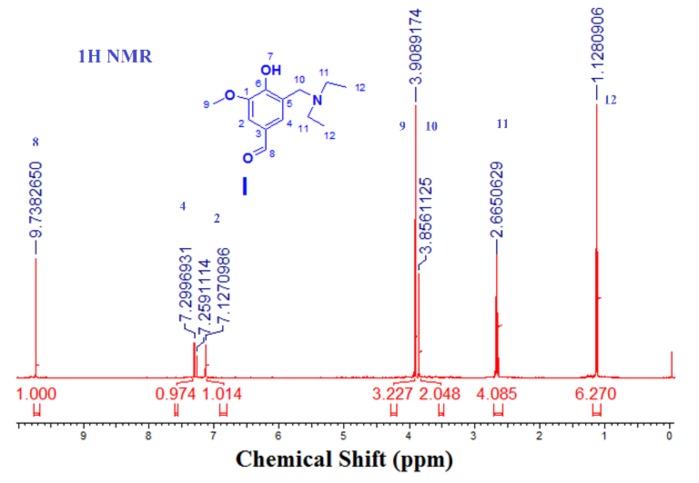
^1^H NMR spectrum (CDCl_3_) of DEAMV.

This has been done by the reaction of vanillin with diethylamine and formaldehyde, according to Mannich reaction mechanism. In this reaction, we did not use any catalysis, especially acid catalysis, which is famously used in Mannich reaction; therefore, protonated tertiary amine has been yielded. This was done by the reaction of vanillin with diethylamine and formaldehyde according to Mannich reaction mechanism. It was chemically evaluated by ^1^H NMR and ^13^C and FT-IR in Figure 1 and Figure 2. All data was in a logic state, and proved the presence of an active aldehyde group at 9.74 ppm and 196 ppm. A tertiary amine group was also detected at 1.13 ppm for 2CH_3_, at 2.66 and 3.85 ppm for 2CH_2_, N(CH_2_)_2_ and Ar–CH_2_–N respectively.

#### 3.2.2. Step 2: Is the formation of 2-[(diethylamino)methyl]-4-formyl-6-methoxyphenyl acrylate (DEMAVA) (**II**)

**Figure 3:**
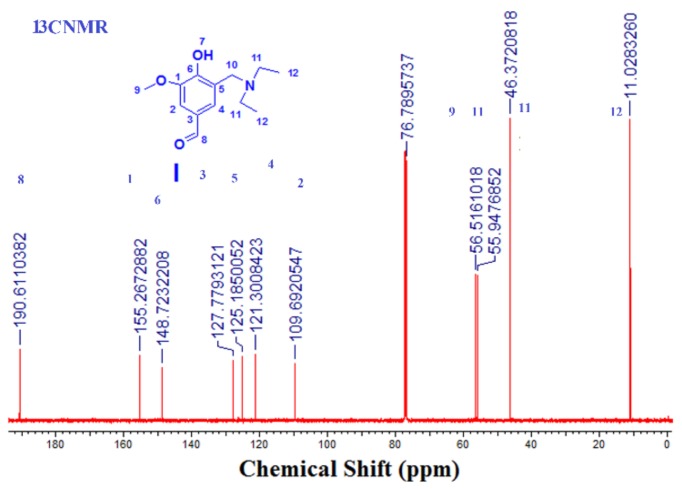
^13^C-NMR spectrum (CDCl_3_) of DEAMV.

**Figure 4:**
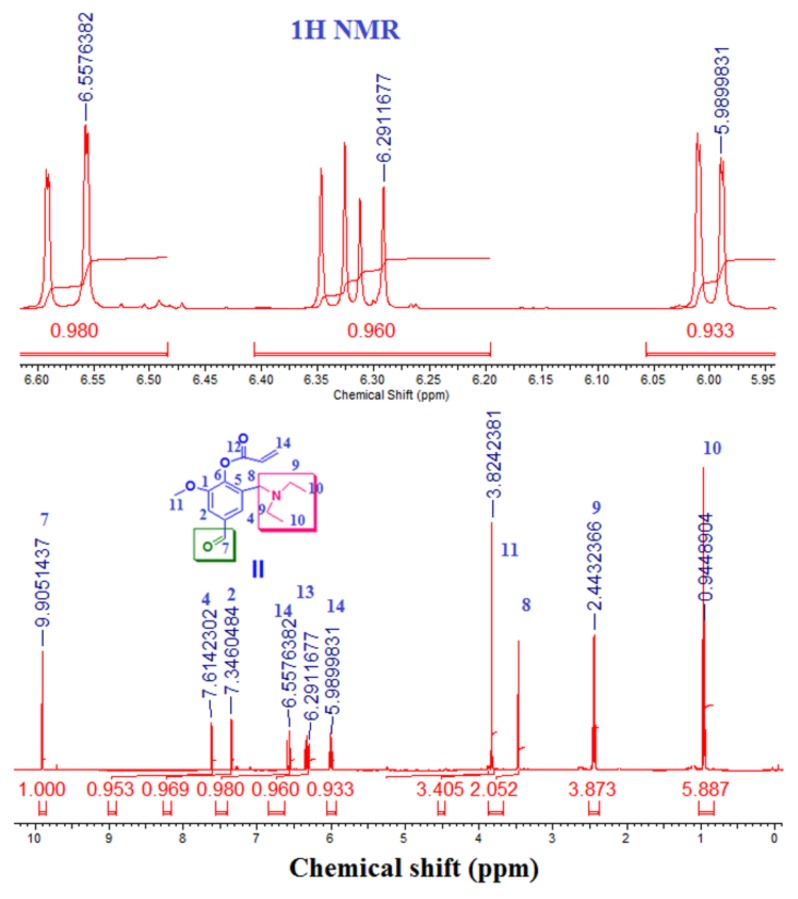
^1^H NMR spectra (CDCl_3_) of DEAMVA.

Compound (**I**) or (3-[(diethylamino) methyl)-4-hydroxy-5-methoxy- benzaldehyde) (DEAMV) was reacted with acryloyl chloride in the presence of triethylamine under the reaction conditions as shown in Figure 1. It was chemically evaluated by ^1^H NMR and ^13^C (Figure 3 and Figure 4) and Fourier-transformed infrared spectroscopy (FT-IR). All data was in a logic state and proved the presence of an active aldehyde group at 9.95 ppm and 192.52 ppm.

**Figure 5:**
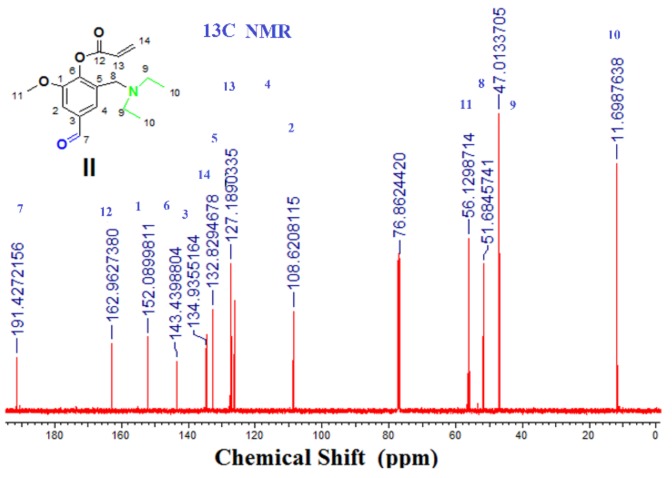
^13^C-NMR spectrum (CDCl_3_) of (DEAMVA).

**Figure 6:**
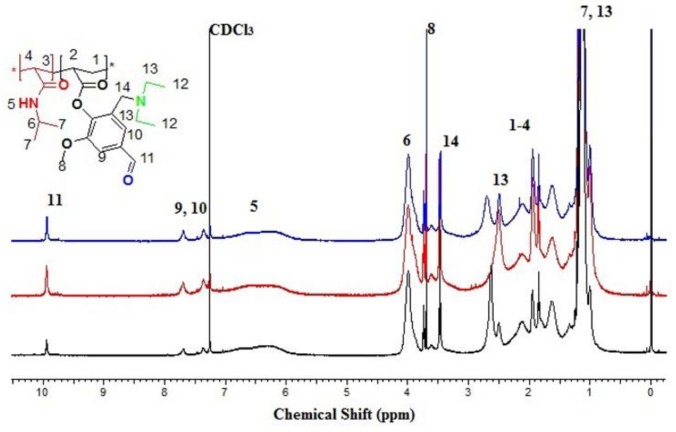
^1^H NMR spectra (CDCL_3_) of poly(NIPAAm-*co*-DEAMVA) with 5, 10, and 15% mole ratio of DEAMVA.

**Figure 7:**
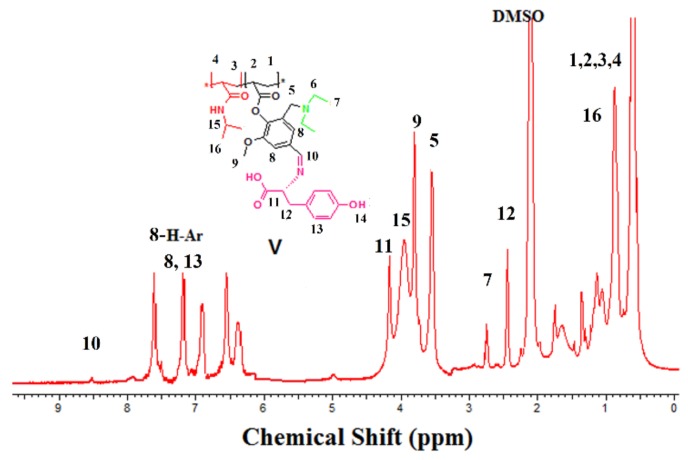
^1^H NMR spectrum (*d*-DMSO) of poly(NIPAAm-*co*-DEAMVA)-*g*-tyrosine.

The chemical reactions for the preparation of dual responsive copolymer with different mole ratios DEAMVA (5, 10, 15% mol) are illustrated in Figure 1. The copolymers were prepared and evaluated by 1HNMR and FTIR, as shown in Figure 5, Figure 6 and Figure 7. The ^1^H-NMR recorded specific signals for **IIIa**–**c** at δ = 0.73–1.35 ppm for 6H dimethyl of NIPAAm, at δ 3.60–3.73 ppm for 3H methoxy group of vanillin, the aromatic protons 2H appeared at δ = 7.08–7.62 ppm, and finally, the most distinguished signal related to the aldehyde group of DEAMVA 1H at δ = 9.73–10 ppm. The FT-IR spectroscopy of polymer with dry KBr, as described in the instrumental part, exhibited signal emphasis of the results of ^1^H NMR. The stretched signals were recorded at 1742 cm^−1^ for carbonyl ester, and at 1650 cm^−1^ for amide. Moreover, the ^1^H NMR was used to determine the actual mole percent of each monomer in the polymer chain. The intensity of the signals at 1.21 ppm of 1H isopropyl group (CH(CH_3_)_2_) was a specific signal of NIPAAm, and 1H at 9.73 ppm of aldehyde (CHO) of DEAMVA, as shown in Table 1.

The grafting of tyrosine and tryptophan into the dual responsive copolymer was enhanced according to Schiff base between the aldehyde group of DEAMVA and the primary amine group of amino acids. The grafting process was done at room temperature in a mixture of ethanol and H_2_O 1:1. Grafted copolymers were elucidated by ^1^H NMR and FT-IR

**Figure 8:**
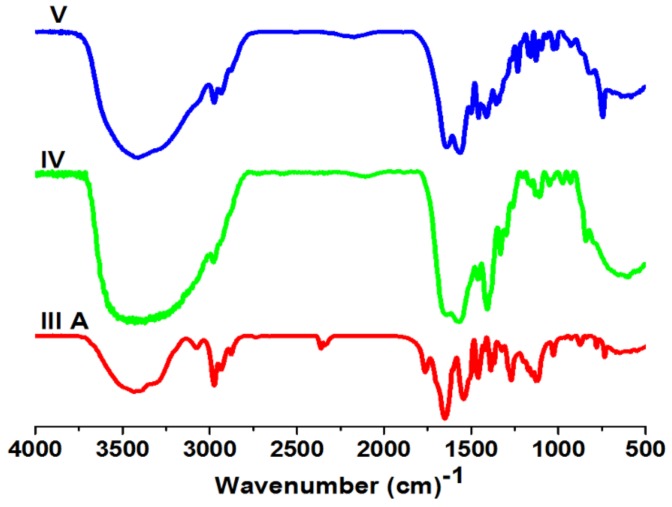
IR spectra and poly(NIPAAm-*co*-DEAMVA) **IIIa–c** of grafted copolymers **IV–V**.

Functionality with the aldehyde group in the polymer main chain served to graft with any amino compound to produce Schiff’s base, as mentioned in reaction Figure 1. The grafting process was done at room temperature in a mixture of ethanol and H_2_O 1:1. Grafted copolymers were elucidated by ^1^H NMR and FT-IR. The evaluation by ^1^H NMR has demonstrated the disappearance of 1H of aldehyde signal at δ = 9.74 ppm and the formation of new functional group imine (HC=N) signal at δ = 8.13 ppm. The FT-IR spectra proved the presence of imine at 1560 cm^−1^, as shown in Figure 7 and Figure 8.

### 3.2. Polymer Characterization

#### Molecular Weight

**Figure 9:**
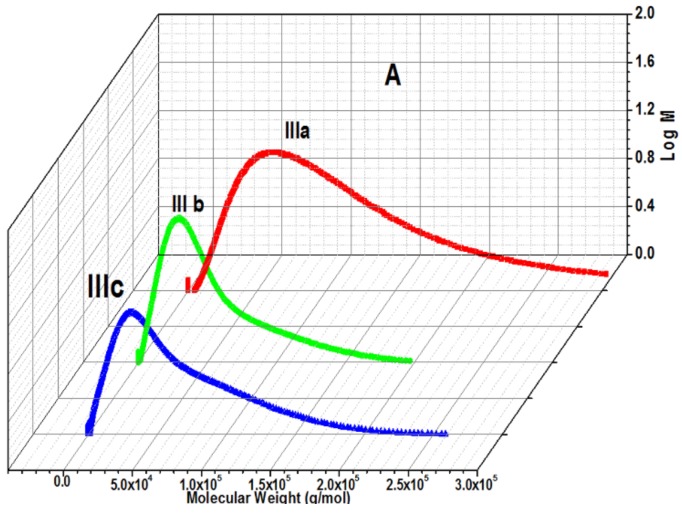

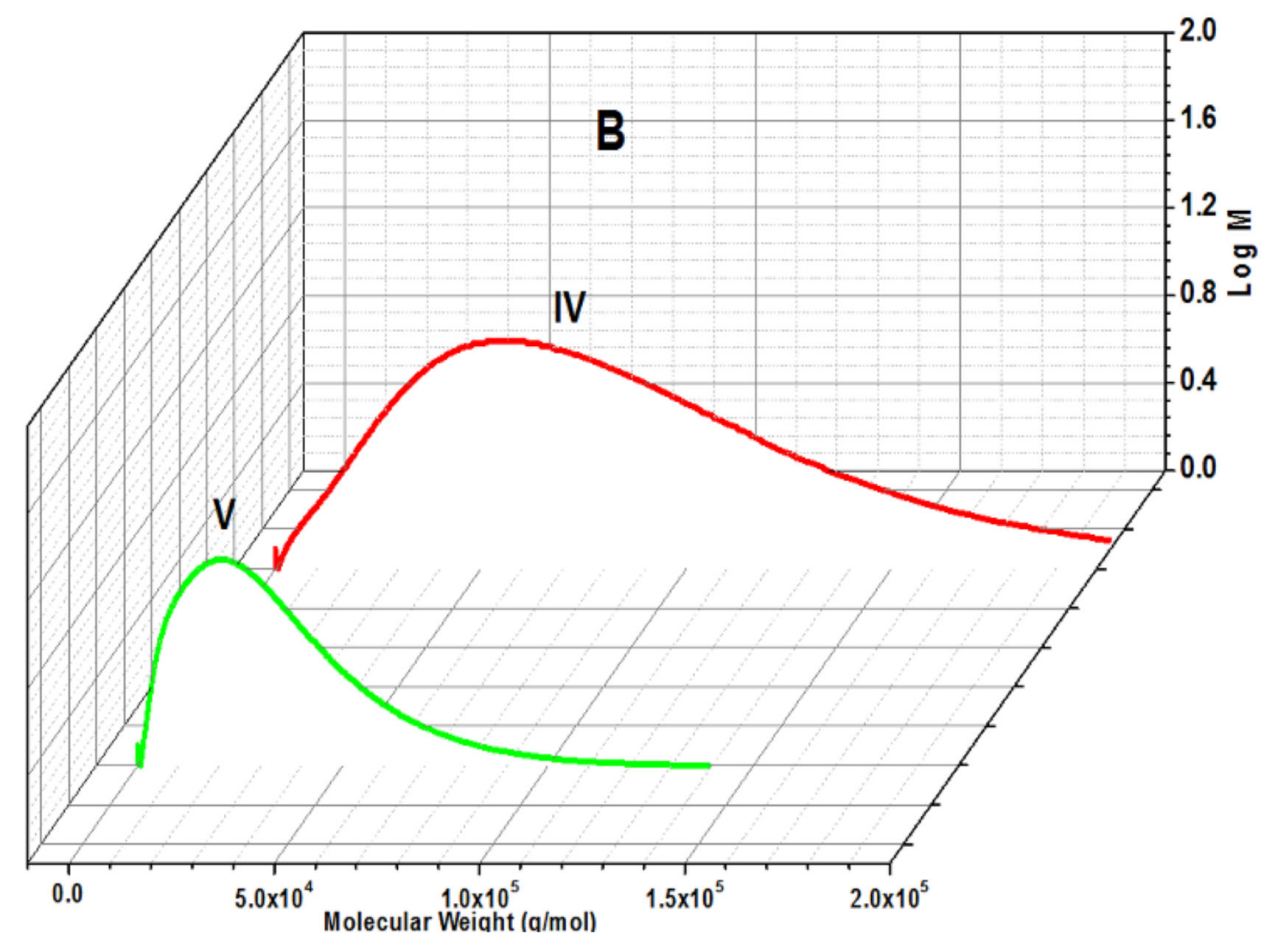
GPC molecular weight of copolymers; (**A**) **IIIa–c**; (**B**) **IV** and **V**.

Molecular weight (M_n_) and molecular weight distribution (M_w_/M_n_) of all polymers, copolymers, and terpolymers were determined by Gel-Permeation Chromatography with Knauer in dimethylacetamide DMAc. Polymer samples (6 g/L) were prepared with 2,6-di-*tert*-butyl-4-methylphenol (BHT) as an internal standard. The measurements were performed at 30 °C. The molecular weight of copolymers and grafted copolymers is summarized in Table 1. The spectra showed one peak, indicating that complete conversion of monomers to the polymer and the absence of low molecular weight and impurities [4,6,29], as shown in Figure 9A,B.

### 3.3. Study of the Phase Separation of Poly (NIPAAm-Co-DEAMVA) and Grafted Polymers

The lower critical solution temperature LCST at pH 1.68 and pH 7 showed an increase as the molar concentration of DEMAVA increased in the polymer chain, as shown in Table 1. This might be due to the formation of charge in the polymer chain causing electrostatic repulsion which further increases the hydrodynamic volume [39,40], as shown in Figure 9. At pH 1.68, the polymer has hydrated tertiary amine and aldehyde groups that converted to OH groups. For this reason, the hydrophilic groups are more effective than the hydrophobic groups of DEMAVA. Hence, overall, the copolymer exhibited higher hydrophilicity.

**Figure 10:**
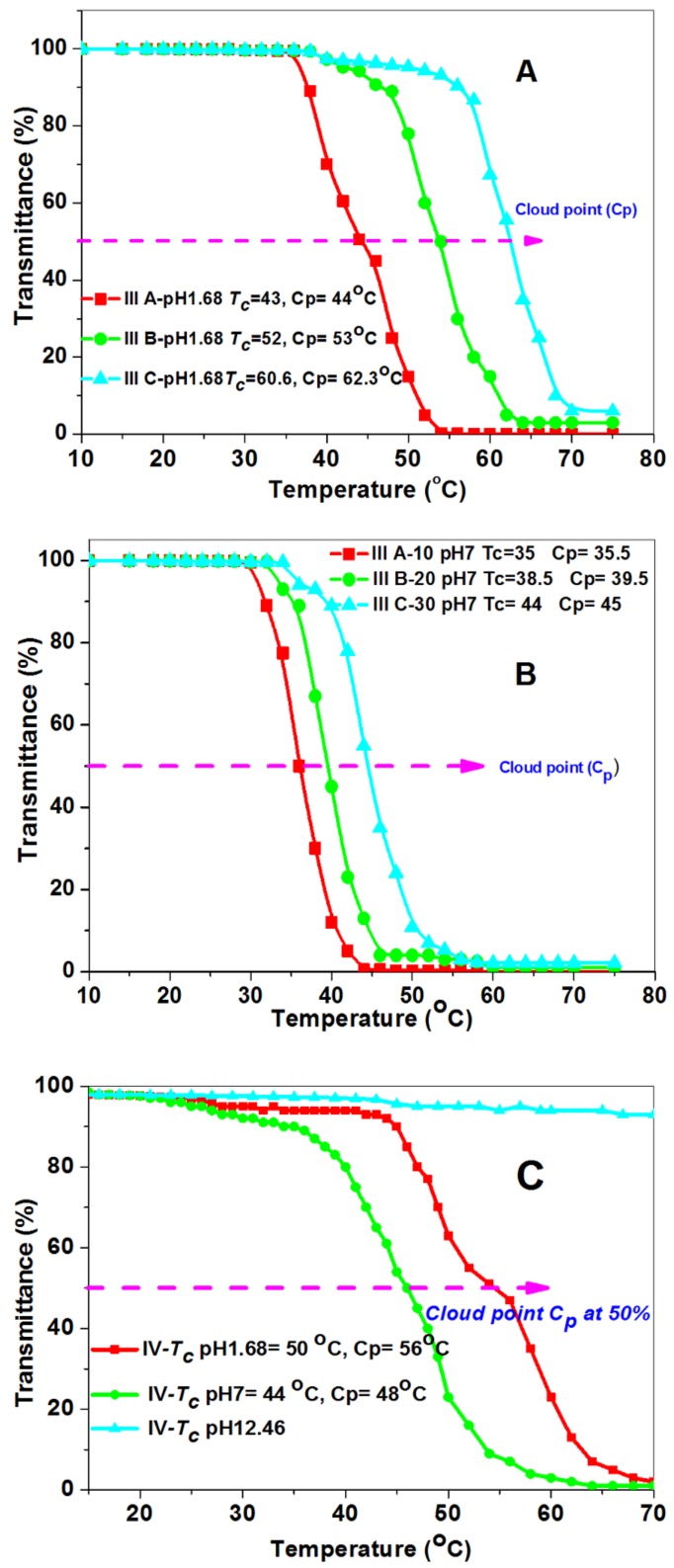

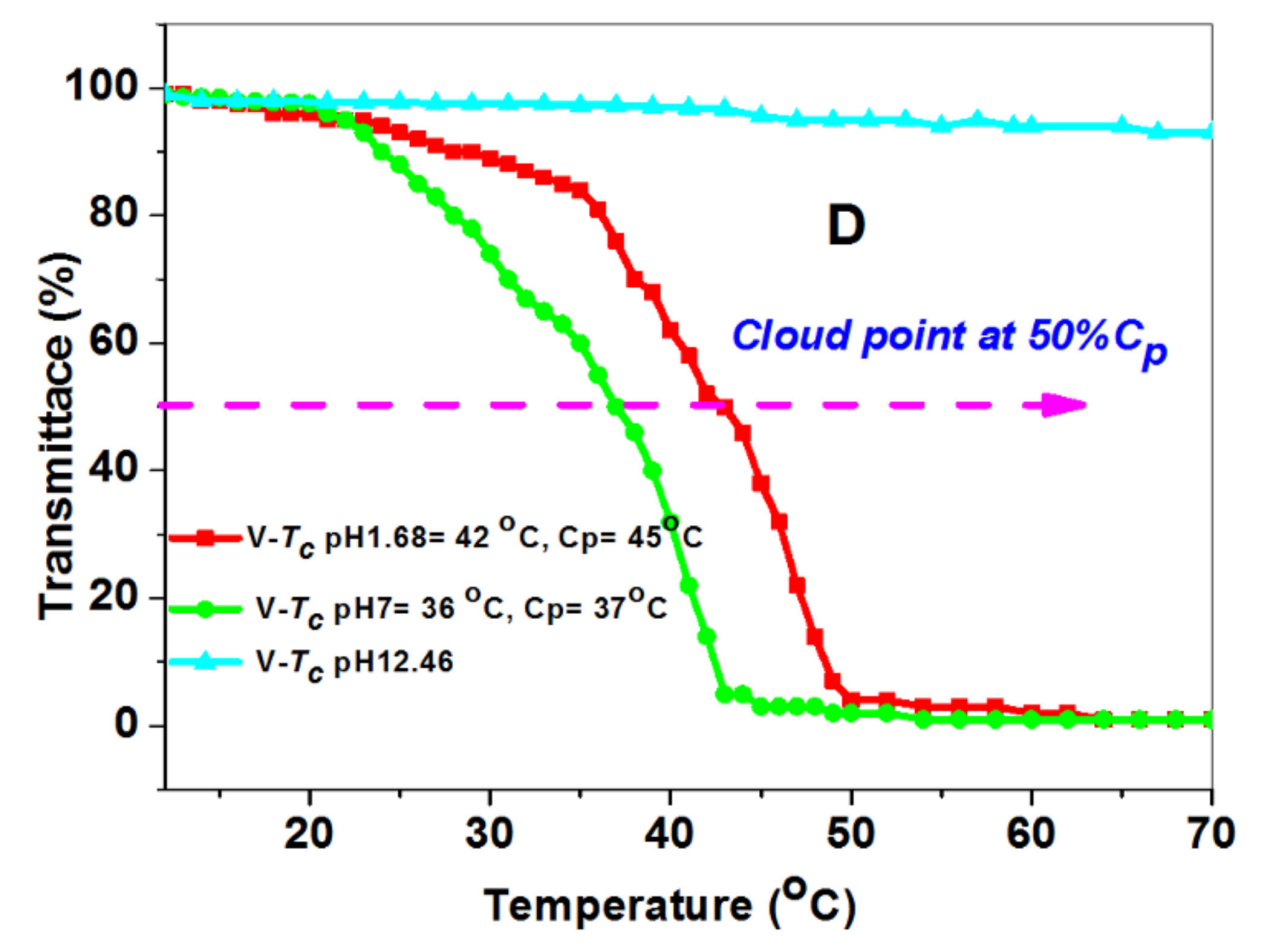
Change of transmittance with temperature (turbidity) for determination the *T_c_* and *C_p_* of P (NIPAAm-*co*-DEAMVA) with different mol % of DEAMVA at pH 1.68, pH 7 and pH 12.46 (**A**,**B**), P (NIPAAm-*co*-DEAMVA)-*g*-tryptophan with 10 mol % of DEAMVA (**C**), and poly (NIPAAm-*co*-DEAMVA)-*g*-tyrosine with 10 mol % of DEAMVA (**D**) using UV-Vis spectroscopy for 1 wt % of polymer solution.

At pH 7, the polymer solution shows no change because the affection of total hydrophilic groups is too weak for DEMAVA. Therefore, the polymer solutions demonstrated LCST which eas closed more to homo-poly (NIPAAm) with respect to the affection of high composition of DEMAVA for polymer **IIIc** with the highest (*T**_c_***) 60.6, 44 °C at pH 2 and pH 7 respectively, as shown in Figure 10A,B.

**Figure 11:**
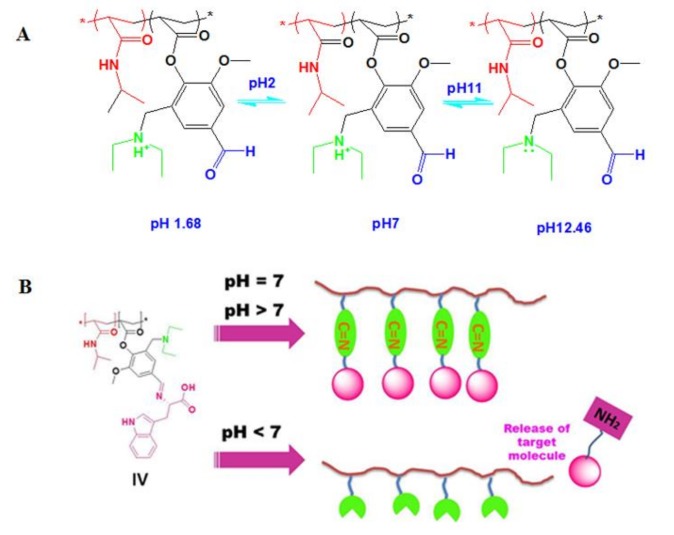
The predicted chemical structures of conjugated copolymer **IV** in different pH solution.

The last feature was detected at pH 11, which did not show any change in LCST starting from 10 °C to 80 °C. For the deprotonating of hydrated tertiary amine and the formation of tertiary amine with a lone pair of electrons as shown in Figure 11, this is responsible for raising LCST to 80 °C.

The post polymerization of poly (NIPAAm-*co*-DEAMVA) with tryptophan and tyrosine **IV** and **V** was achieved by chemical reaction with the formation of a Schiff base, which showed responsiveness to pH addition to the presence of tertiary amine group.

**Figure 12:**
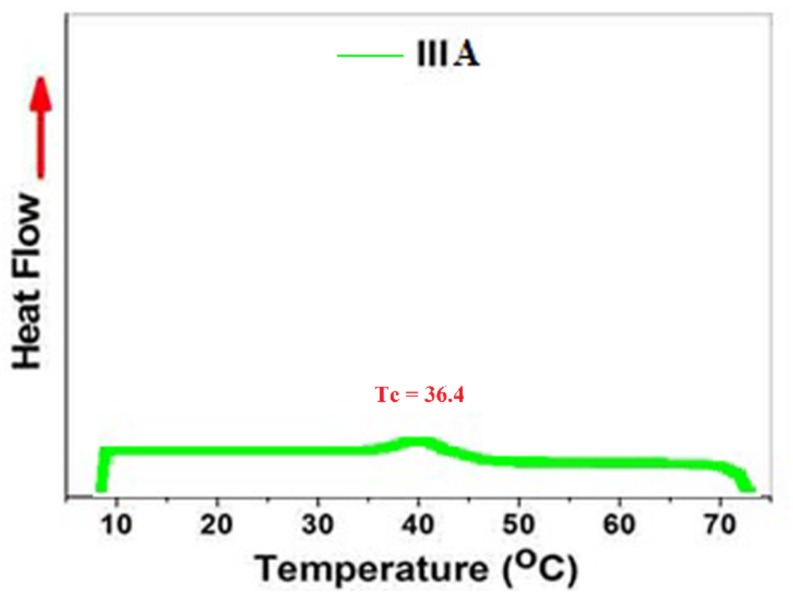
Micro-DSC of polymer solution (**IIIc**) for determination of LCST (*T_c_*).

The conjugation of tryptophan and tyrosine with polymer molecules is based on the formation of a Schiff base through the reaction of amine group with aldehyde and the formation of imine. This kind of molecule is stable in alkaline conditions, and decomposed in stronger acidic one. For this reason, it characterizes as a stimuli-responsive linker in polymer chemistry [41]. The imine group affects the electrical charge in the polymer chain. After conjugation, the copolymers became more hydrophilic than at first, and gave a dual response character to pH and temperature. The lower critical solution temperature of the conjugated polymer solution was measured by UVVis-spectroscopy in different pH buffer solutions (pH 1.68, pH 7 and pH 12.46). For copolymer **IV** and **V** conjugated with tryptophan and tyrosine respectively, we observed a rise in *T_cs_* in pH 1.68 to more than pH 7, due to the release of amino acid and the formation of charge along the polymer chain. The other observation was the higher ***T_c_*** value for the conjugated copolymer **IV**, with tryptophan at pH 1.68 exhibiting ***T_c_*** = 50 °C compared to the ***T_c_*** value for the conjugated copolymer V with tyrosine ***T_c_*** = 42 °C, indicating the higher hydrophilicity of **IV** compared to **V**, according to the chemical structure of the conjugated amino acid, which proves the higher hydrophilicity of tryptophan compared to tyrosine [42]. As previously noted, the ***T_c_*** at stronger basic conditions, i.e., pH 12.46, could not be recorded until the highest temperature, 80 °C, due to the greater electrical charge in the polymer chain. The lower critical solution temperature *T_c_* was taken as the inflected point for all copolymers further. The cloud point was also detected at 50% transmittance. Table 1 summarizes all values. The (***T_c_^,^s***), Figure 10A–D showed a relationship between temperature and transmittance. All the *T_c_* values for copolymers were calculated as the inflected point, while the cloud point was taken at 50% transmittance Micro-DSC, and was also used to determine the ***T_c_***of copolymer **IIIa** in pH 7. The different ***T_c_^,^s***values have been detected by UV-Vis. Spectroscopy and micro-DSC Figure 12, depended on the definitions of the *T_c_* as the inflected point or the onset respectively [43].

### 3.4. Determination of the Conversion of poly (NIPAAm-Co-DEAMVA) to Poly (NIPAAm-Co-DEAMVA)-g-Tryptophan

**Figure 13:**
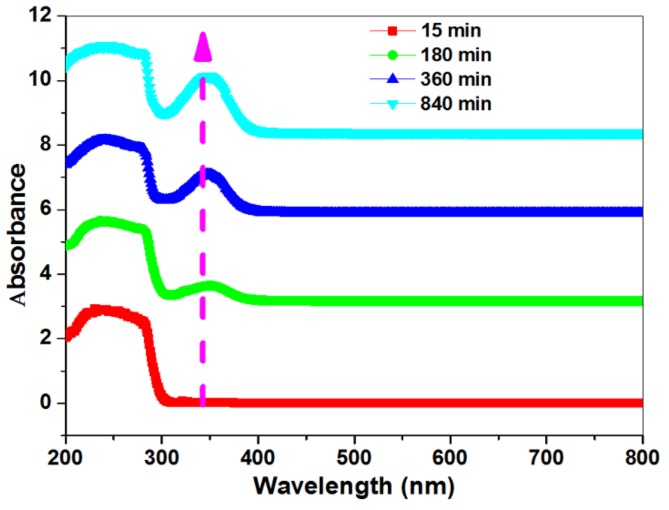
UV-vis. Spectroscopy for the formation of grafted poly (NIPAAm-*Co*-DEAMVA)-*g*-tryptophan as a function of absorbance with time.

After the post polymerization and conjugation with amino acid, it was important to determine the conversion of poly (NIPAAm-*co*-DEAMVA) to the grafted one. For this purpose, we used poly (NIPAAm-*co*-DEAMVA)-*g*-tryptophan for the experiment. The conversion was related to time. UV.vis. Spectroscopy was used to record the conversion at the different times (15, 180, 360, and 840 min) by recording the absorption for each run, as shown in Figure 13. The Labert-Beer law has been used for to determine the concentrations and mol % of conversion. The absorption peaks exhibit the disappearance of the absorption of carbonyl aldehyde at 250–270 nm for n-π*, π-π* and a good appearance of the new peaks at 340–380 related to C=N imine linkage n-π*. As is clear in Figure 12, the C=N absorption increased steeply with time to get the greatest value at 840 min. Table 1 shows the conversion percentages at 840 min for poly (NIPAAm-*co*-DEAMVA)-*g*-tryptophan and poly (NIPAAm-*co*-DEAMVA)-*g*-tyrosine.

### 3.5. The Thermal Properties

**Figure 14:**
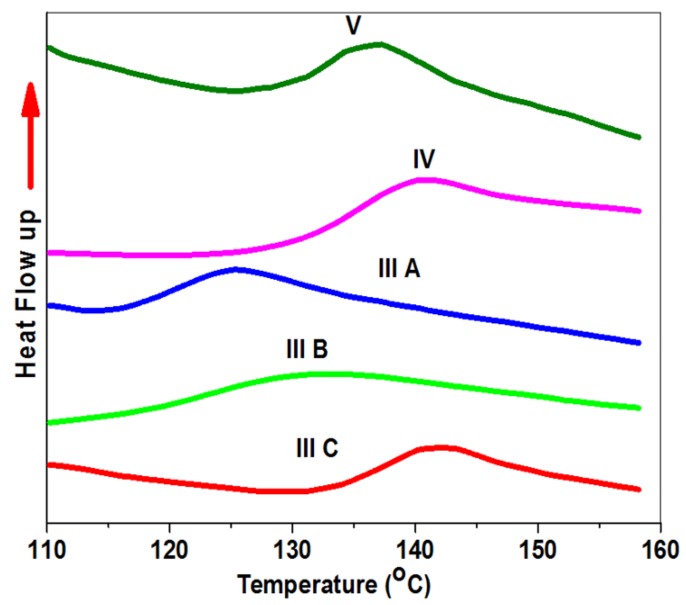
DSC thermogram of copolymers and grafted copolymers for determination of glass transition temperature (***T_g_***).

The glass transition temperature ***T_g_*** is the most important thermal property. A Differential Scanning Calorimeter was used for recoding ***T_g_*** for all polymers. The measurements were taken for dry samples at a heating rate 5 °C/min at the midpoint inflection of the thermogram. The thermogram exhibits one ***T_g_*** value, proving random copolymerization [44]. A comparison of the ***T_g_^,^s*** values of PNIPAAm homopolymer, copolymer with DEAMVA and grafted copolymers with tryptophan and tyrosine, was undertaken. Table 1 summarizes the (*T_g_^,^s)* values. Firstly, a recent study measured the glass transition temperature of homo-PNIPAAm ***T_g_*** at 135 °C [44]. The incorporation of DEAMVA with its hydrophilic and hydrophobic groups in the PNIPAm main chain will shift the ***T_g_*** values to 122, 127, and 137 °C for **IIIa, IIIb,** and **IIIc** respectively. It has been demonstrated in Figure 14 that an increase in the *T_g_* values occurs by increasing the molar concentration of DEAMVA; this is attributed to the increase in hydrophilicity, which leads to a decrease in the spacing and lower the chain interaction lower in the flexibility [45]. After the conjugation of the copolymer with tryptophan and tyrosine, we noticed an increase in ***T_g_*** due to the same factors discussed previously; in spite of this, the ***T_g_*** of grafted copolymer with tryptophan **IV** has a smaller increase in ***T_g_*** 136 °C than tyrosine **V** 133 °C for the higher hydrophilicity of tryptophan than tyrosine, supporting from its chemical structure.

### 3.6. Morphological Studies

**Figure 15:**
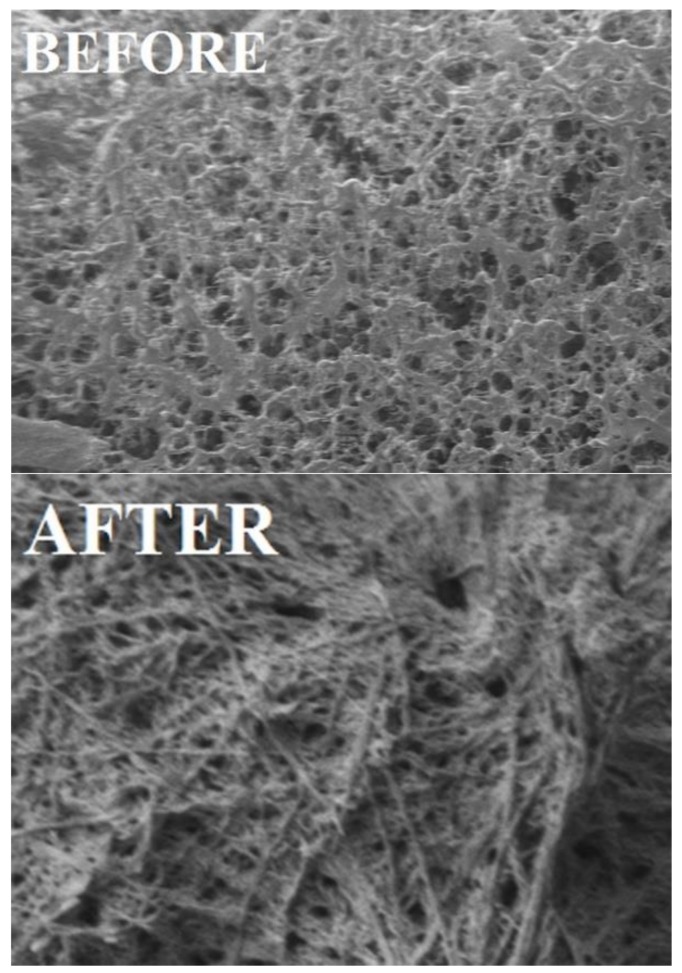
SEM images of copolymer **IIIb** (before grafting) and after grafted copolymer with tyrosine V at 500× magnification.

The morphology of solid polymers has been distinguished by a scanning electron microscope (SEM). The scanning was done for copolymer **IIIb** before and after grafting the copolymer with tyrosine **V**. Figure 15 is the SEM photograph before and after grafting at a magnification of 500×. The polymer surface before grafting seems smooth and compact, with small pores. After grafting with tyrosine, it appears as a waxy and coarse surface with some cross-links; these morphological changes were due to the grafting of tyrosine in the copolymer chains.

## 4. Conclusions

Vanillin acrylate monomer has been prepared in two steps; first, by the preparation of vanillin with tertiary amine group which then reacted with acryloyl chloride for the preparation of the final product, DEAMVA. This monomer was distinguished by its stimuli-responsiveness to pH, in addition to its functionality. In the preparation of thermal and a pH dual responsive polymer, we used *N*-isopropyl acrylamide (NIPAAm) with three different molar concentrations of DEAMVA, using a free radical polymerization technique. All chemical evaluation of monomers and polymers were in a logic state, and proved the presence of aldehyde groups, which were used in the grafting of amino acid. Due to the thermal and pH-responsive behavior of the polymer, the phase separation and LCST are of great interest. The LCST of copolymers showed higher ***T_c_*** than PNIAAm for its higher hydrophilicity, and exhibited a regular increasing rate with the molar concentration of DEAMVA. The grafting of amino acid was facilitated by the presence of the aldehyde group in the polymer chain. The grafting process occurred with tryptophan and tyrosine; both were evaluated by chemical methods and proved the disappearance of aldehyde group and the formation of imine. The lower critical solution temperature LCST and ***T_c_*** was recorded and showed an increased value for tryptophan over tyrosine. The conversion was also studied as a factor with time, demonstrating the highest conversion at 840 min. DSC thermogram for copolymers and grafted copolymers showed higher ***T_g_*** values by increasing the concentration of DEAMVA. Moreover, the glass transition temperature of the grafted tryptophan was higher than that of the grafted tyrosine. SEM morphological photograph of the grafted copolymer showed some cross-links, which were attributed to the grafted molecule.

## Figures and Tables

**Table 1:** Yield, composition, conversion, average molecular weight, polydisperisity, and glass temperature of P(NIPAAm-*co*-DEAMVA) 5, 10, 15% mole ratio of DEAMVA and grafted Poly(NIPAAm-*co*-DEAMVA).

Polymer	Yield (%)	^1^H NMR DEAMVA (mol %)	Conversion (%)	Mn ^a^ (g/mol) 10^4^	Ð ^b^	*T_g_*^c^ (^°^C)	*T_c_*^d^ (°C)		
							pH 7	pH 1.68	pH 12.46
**IIIa**	83	3.55	-	15,340	1.98	122	35	43	-
**IIIb**	82	7.26	-	12,260	2.27	127	38.5	52	-
**IIIc**	76	11.36	-	10,580	2.46	137	44	60	-
**IV**	80	-	84	7750	1.87	136	50	44	-
**V**	81	-	79	10,270	2.12	133	42	42	-

^a^ Number average molecular weight, ^b^ Polydispersity, ^c^ Glass transition temperature, ^d^ Lower critical solution temperature.
